# From Paper to Digital Applications of the Pain Drawing: Systematic Review of Methodological Milestones

**DOI:** 10.2196/14569

**Published:** 2019-09-05

**Authors:** Nour Shaballout, Till-Ansgar Neubert, Shellie Boudreau, Florian Beissner

**Affiliations:** 1 Somatosensory and Autonomic Therapy Research Institute for Neuroradiology Hannover Medical School Hannover Germany; 2 Department of Health Science and Technology Aalborg University Aalborg Denmark

**Keywords:** pain drawing, digital pain drawing, pain chart, pain map, pain body map, pain diagram, ehealth, medical app

## Abstract

**Background:**

In a pain drawing (PD), the patient shades or marks painful areas on an illustration of the human body. This simple yet powerful tool captures essential aspects of the subjective pain experience, such as localization, intensity, and distribution of pain, and enables the extraction of meaningful information, such as pain area, widespreadness, and segmental pattern. Starting as a simple pen-on-paper tool, PDs are now sophisticated digital health applications paving the way for many new and exciting basic translational and clinical applications.

**Objective:**

Grasping the full potential of digital PDs and laying the groundwork for future medical PD apps requires an understanding of the methodological developments that have shaped our current understanding of uses and design. This review presents methodological milestones in the development of both pen-on-paper and digital PDs, thereby offering insight into future possibilities created by the transition from paper to digital.

**Methods:**

We conducted a systematic literature search covering *PD acquisition*, *conception of PDs*, *PD analysis*, and *PD visualization.*

**Results:**

The literature search yielded 435 potentially relevant papers, from which 53 methodological milestones were identified. These milestones include, for example, the grid method to quantify pain area, the pain-frequency maps, and the use of artificial neural networks to facilitate diagnosis.

**Conclusions:**

Digital technologies have had a significant influence on the evolution of PDs, whereas their versatility is leading to ever new applications in the field of medical apps and beyond. In this process, however, there is a clear need for better standardization and a re-evaluation of methodological and technical limitations that no longer apply today.

## Introduction

Pain is a multifaceted subjective experience that poses unique challenges for objective assessment [1]. To date, many qualitative and quantitative assessments of pain rely solely on self-reporting. Perhaps, the simplest method involves pointing to the painful area or the use of words to describe the location and, if known, the quality of the pain. However, pointing and use of words often lack clarity and are challenging to quantify. A more objective tool for capturing pain location and even quality, amongst other aspects of the subjective pain experience, is a pain drawing (PD). When using a traditional PD, the patient marks or shades the location of pain and related symptoms on an outline of the human body or parts thereof [2,3]. This form of communication allows physicians to capture the intensity, localization, and distribution characteristics of a patient’s pain experience and extract meaningful and quantifiable information, such as pain area, intensity, and widespreadness.

Starting in 1949 as a simple pen-on-paper tool [2], PDs evolved into electronic form by the 1990s [4-9]. This evolution is now paving the way for new and exciting, basic translational and clinical applications. However, to grasp the full potential of digital PDs, it is necessary to understand and learn from the historical evolution of PDs and the methodological developments that have shaped our current understanding of uses and design. Furthermore, a consideration of the available digital technologies to date necessitates a re-evaluation of methodological and technical applications of PDs.

Previous reviews addressed specific aspects of PDs, such as iconography [3], reproducibility and reliability [10], association with psychological factors [11], and suitability for psychological screening [12-14]. This comprehensive review serves to assimilate the innovations and methodological milestones over the last 70 years that have advanced and shaped clinical and scientific application. Knowing these milestones is essential for the design of future PD applications in the context of mobile health. A further aim of the literature review was to uncover and reveal potentially overlooked and forgotten milestones related to *PD acquisition*, *conception of PDs*, *PD analysis*, and *PD visualization.*

For this review, *PD acquisition milestones* are defined as changes in the way we collect PDs in the clinic or research setting. *Conceptual milestones* represent advancements in our understanding of what information PDs can capture and the value this information provides. *PD analysis milestones* are new methods or approaches developed for extracting clinically relevant information, whereas *PD visualization milestones* are innovative designs or techniques for conveying meaning and presenting the information captured by the PD.

## Methods

Given that PDs are also known as pain charts [2], pain maps [15], pain body maps [7], or pain diagrams [16], these terms in their singular and plural forms formed the basis of the search. To account for new advancements, we further added the term *digital* to identify this new form of PDs specifically. A systematic literature search in PubMed [17] using these terms was performed as of September 16, 2018. From this time, it yielded 512 results, and an additional 24 publications were further identified from reference lists of the initial search results and additional Web searches on Google scholar [18].

In a first pass, abstracts and (if necessary) text bodies of all publications were screened by authors NS and TN to check for the following exclusion criteria: (1) PDs were not based on body templates or parts thereof, (2) PDs were not made by patients or test subjects but by physicians or investigators, (3) study results were not obtained in adults, (4) the publication was a review article, and (5) the publication was not in English. This resulted in the exclusion of 101 publications. All remaining 435 papers were considered for further review, and the abstract and text body were screened by authors NS and TN in a second pass to identify papers that first disseminated potential methodological milestones.

**Figure 1:**
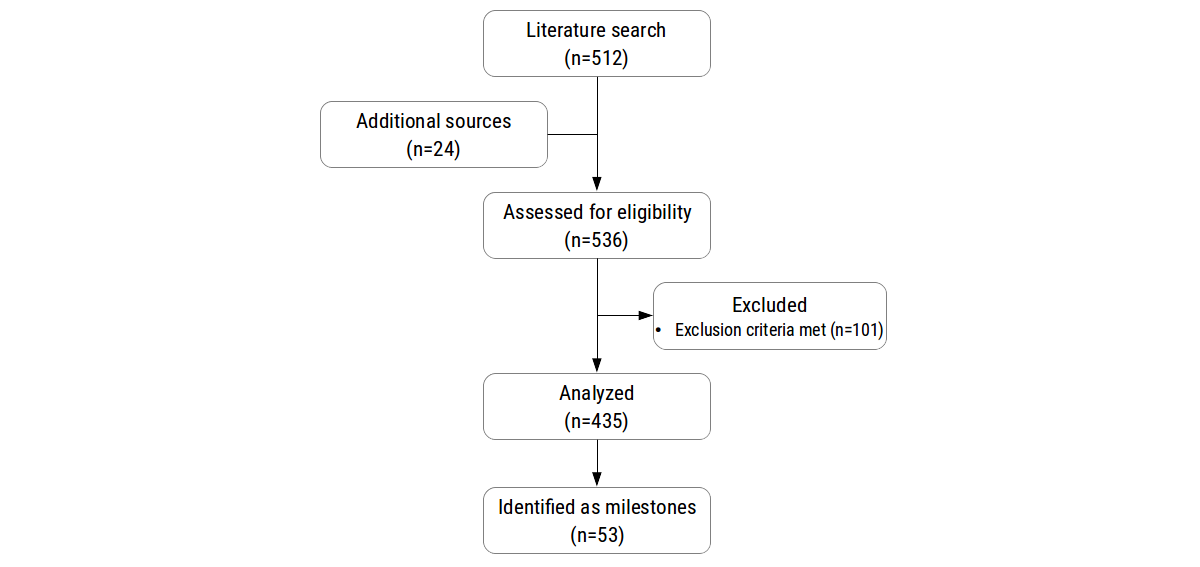
Flow-chart of the literature search. Of the 53 methodological milestones identified by our search, 19 described pain drawing (PD) acquisition milestones, 18 conceptual milestones, 31 for PD analysis milestones, and 4 PD visualization milestones.

**Figure 2:**
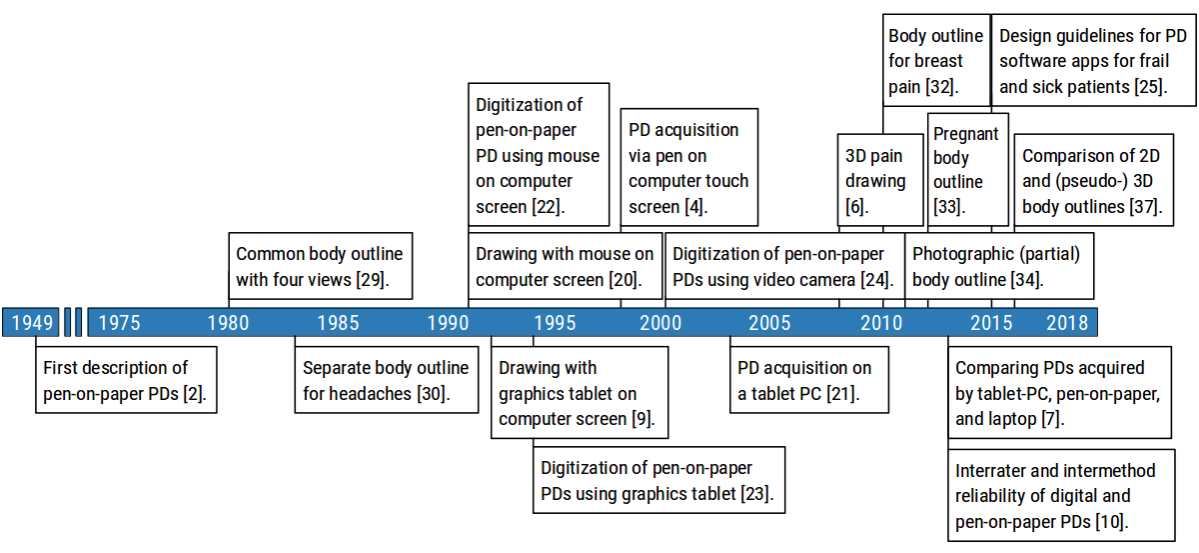
Methodological milestones in the area of PD acquisition. The acquisition methods for acquiring PDs over the last 2 decades appear to mirror the commercialization of digital technologies. PD: pain drawing; PC: personal computer; 3D: three-dimensional; 2D: two-dimensional.

In a third and final pass, papers constituting potential methodological milestones were reviewed by authors NS, TN, and FB. All potential milestones were followed up by performing a literature search to confirm provenance and rule out previous description by other papers. The final list of all milestone papers was reviewed and accepted by all authors. A flowchart of these procedures is shown in Figure 1 and the milestones have been illustrated in Figures 2, 4, 6, and 7.

## Results

### Brief Overview

We identified 53 milestone papers, of which 19 described PD acquisition milestones, 18 conceptual milestones, 31 PD analysis milestones, and 4 PD visualization milestones (see Multimedia Appendix 1).

The following sections discuss, together with other relevant scientific findings supporting or expanding, the significance of the identified milestones. Some of the milestones belong in more than one category and therefore are discussed where appropriate.

### Pain Drawing Acquisition Milestones

PD acquisition milestones can be separated into 2 main topic clusters: PD data collection and digitization, and body templates.

#### Data Collection and Digitization

Albrecht Dürer’s Renaissance drawing *The Sick Dürer* may be the first recorded account of a PD [19]. The first modern PDs, however, were pen-on-paper drawings where the patient used a pencil to “mark in on the charts wherever he experiences pain” [2]. Owing to its simplicity and ubiquity, the pen-on-paper acquisition method is probably the most common to date and will likely continue until supporting digital technologies become more widely adopted.

In 1991, Mann et al were first to acquire PDs directly on a computer [20] to assess the potential of using artificial neural networks (ANNs) for diagnosing low back pain disorders. However, pixel-based counts were first performed by North et al using a graphics tablet (Kaola) connected to an ordinary IBM personal computer (PC) to explore paresthesias evoked by implanted neurological stimulators [9]. For similar purposes, Aló et al acquired PDs with the aid of a pen-based interactive computer screen using a Windows-based software program (PainDoc, Quest-ANS Inc) [4]. A few years later, the same concept was replicated on a tablet PC (PenCentra 200, Fujitsu, Inc) [21] and tested as a clinical tool in a randomized control trial for automatic adjustments of a spinal cord stimulator.

The introduction of digitizing pads, electronic cameras, and image scanners facilitated computer-aided analyses of PDs by transforming pen-on-paper drawings into a digital format. Here, Mann et al were the first to digitize patients’ pen-on-paper PDs by manually redrawing them on a computer screen using a mouse [22]. Bryner [23] in 1994 used a digitizing pad together with a custom-made software program written in Visual Basic (Microsoft) to create a digital PD (62,102 pixels) to quantify and compare the sensitivity for assessing pain extent between manual grid-based approaches and pixel-based counts. A unique approach to digitize the PD involved using a camera to capture an image of pen-on-paper PD to utilize ANNs [24]. Subsequent advancements from this point forward are difficult to assess as details on the exact digitization process are often insufficiently described in the published methods.

There are several studies focusing on assessing the clinical utility of PDs, and now, with the introduction of digital PDs, the usability and reliability of these platforms needed validation. Southerst et al were the first to assess and show good-to-excellent interrater and intermethod reliability of digitally acquired information about pain distribution to that obtained using pen-on-paper PDs [10]. In the same year, Jaatun et al assessed user interactions with paper, computer, and PC tablet drawings and reported a preference for tablet PDs, as based on patient opinions [7]. Two years later, the same authors published the first set of guidelines for designing PD software interfaces for patients with physical or cognitive impairments [25]. In 2016, the first quantitative comparison between paper and a digital platform (tablet PC) to collect PDs showed a high level of consistency and agreement [9].

In summary, the transitions for acquiring PDs over the last two decades appear to mirror the commercialization of digital technologies such as graphics tablets, touch screens, and custom-made computer programs. However, the driving forces for implementing these new technologies are to facilitate or automate methods for treating, diagnosing, or managing pain. Collectively, the assessment and results of these acquisition milestones suggest that clinicians and researchers may choose either medium for acquiring PDs and can expect to see more digital PD technology in the future.

#### Body Templates

**Figure 3:**
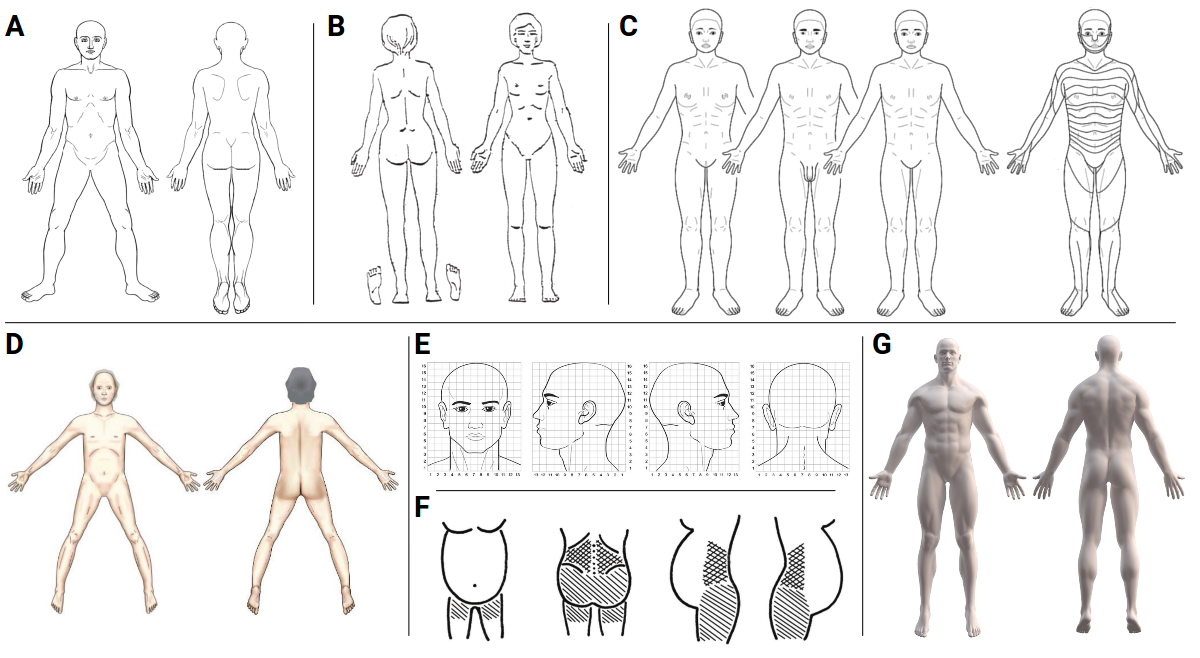
The body template is a crucial ingredient of every pain drawing (PD) and should be chosen carefully as it may influence how much a patient is able to identify with the depicted body and thus impact the quality of the PD. (A) Body outline used by Palmer and many other early publications (modified after [28]). (B) Female body template (modified after [26]). (C) Hannover Body Template, a free body template with dermatome data (under CC BY 4.0) [87]. (D) Body template for frail and very sick patients (under CC BY-NC-ND 4.0) [27]. (E) Partial body template for the depiction of headaches (under CC BY 3.0) [57]. (F) Pregnant body template (under CC BY 3.0) [33]. (G) Pseudo-three-dimensional body template [5].

The reliability and accuracy of PD data collection methods for acquiring pain area, extent, and distribution are also highly dependent on the body template (or manikin). The literature search revealed several different body templates, as shown in Figure 3 [26,27]. Indeed, the body template is the central component of every PD. The features portrayed in the body template may influence a patient’s ability to identify with the body and impact the quality of the PD.

The works of Palmer used an outline as body template, see Figure 3, that had already been in use for at least 50 years in the medical literature [2]. The exact origin of the body template outline is unclear. However, the body template was used in the seminal work by Henry Head on referred pain in visceral disease [28] in which the author made the drawings himself. Almost a century later, Margoles (1980) wrote a letter to the editor-in-chief of PAIN, requesting the use of a standard body template for the purpose of harmonizing PDs for accurate comparisons [29]. The proposed body template outline consisted of an anterior, posterior, and lateral views as well as the soles of the feet. However, this request was not widely adopted as evident by numerous versions of body templates in the scientific literature and clinical settings.

A body outline with additional views of the right and left head and upper and lower jaws for better depiction of orofacial pain, such as headaches and toothaches, was introduced by Toomey et al in 1983 [30]. Udén and Landin recognized the demand for sex-specific body outlines [31] and introduced the first body outline for female patients. This was naturally followed by the first body outline for patients with breast pain [32] and the first pregnant body outline [33]. Most recent are body templates depicting a realistic actual self [34,35]. The first realistic depiction used for basic and clinical research purposes is a photograph body templates and three-dimensional (3D) and pseudo-3D body templates. The first 3D body template for PDs was introduced by Ghinea et al in 2008 [6]. More realistic 3D and pseudo-3D body representations were later developed by other groups [5,36,37]. Comparing two-dimensional (2D) male and female as well as matching 2D and (pseudo-)3D body templates Egsgaard et al found that a majority of patients preferred sex-specific body templates and recommended 2D and (pseudo-)3D body templates to be used according to patient’s preferences [37].

### Conceptual Milestones

**Figure 4:**
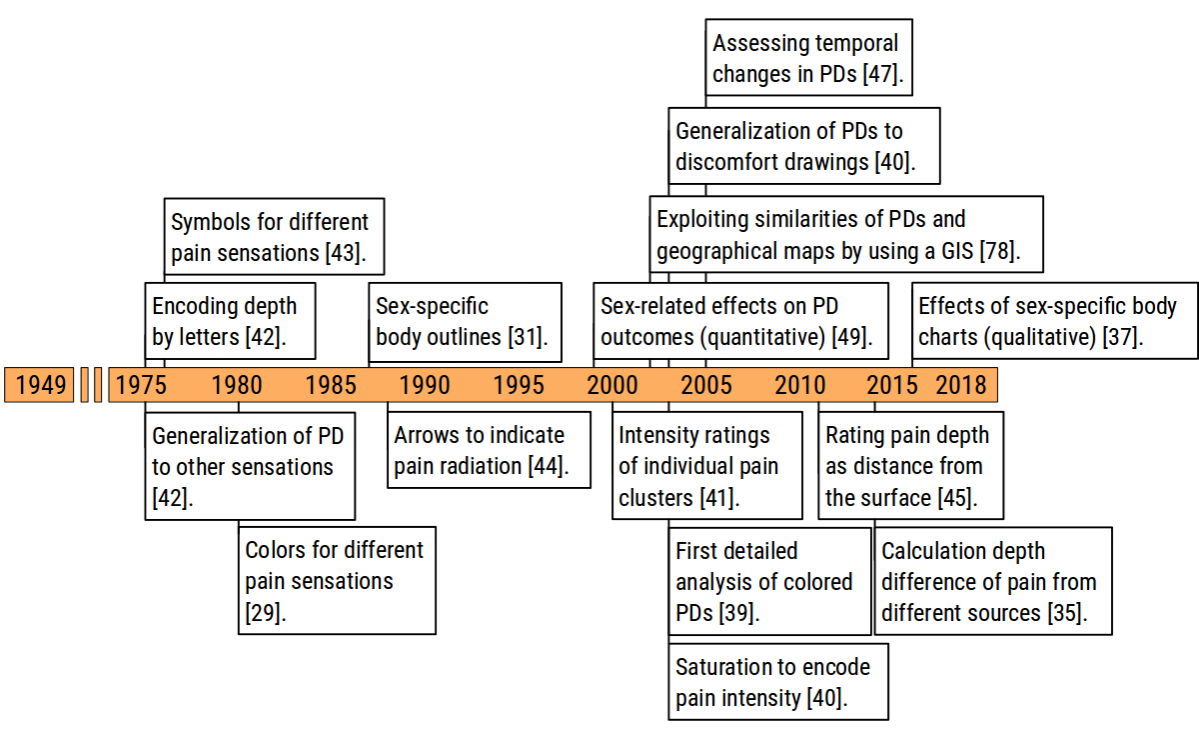
Methodological milestones in the area of conceptual pain drawing developments. PD: pain drawing; GIS: geographical information system.

Conceptual milestones are ideas or results that have advanced our fundamental understanding of what PDs represent and what we can achieve by implementing them. A historical timeline review of these milestones is outlined in Figure 4. We identified 3 clusters of conceptual milestones: elements, generalizations, and sex-specific aspects of the PD.

#### Elements of the Pain Drawing

The largest of 3 conceptual milestone clusters addresses the typical building blocks or elements of PDs.

**Figure 5:**
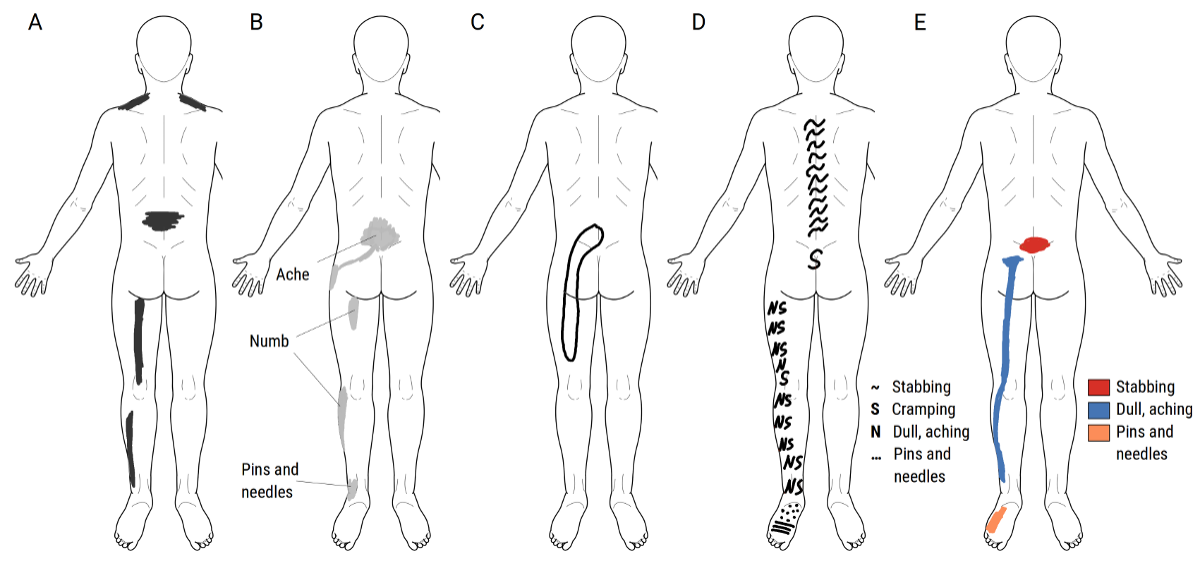
Schematic illustration of common methods for encoding pain location and sensation type in a pain drawing (PD). (A) Marking painful regions. (B) Tracing the outline of painful regions. (C) Marking painful regions and adding annotations. (D) Filling painful regions with predefined symbols. (E) Marking painful regions in predefined colors. All drawings were recreated using the app SymptomMapper [80], developed at Somatosensory and Autonomic Therapy Research, Hannover Medical School, and all methods are currently in use in both digital and pen-on-paper PDs.

The idea of using color to represent different pain qualities or sensations, as illustrated in Figure 5, is well known but the exact origin less easily traced. Palmer wrote that after patients completed their PDs, he asked them about their type of pain and added this information to the PD himself [2]. The first clear documentation of using colors was presented by Margoles in the 1980s [29,38] and adopted by many in subsequent years. However, the first detailed analysis of the colored PDs emerged in 2003, when Masferrer et al reported “that colored pain drawings are no less useful than the black and white approach” [39]. Although pencils enable the encoding of pain intensity by shades of grey or color (eg, darker shades representing stronger pain), the applicability was not systematically exploited until Bertilson et al included the following instructions: “Shadow all pain/discomfort [...], shadow darker where there has been more discomfort” [40]. Before that, Türp et al introduced pain intensity ratings of individual clusters in a study exploring how generic pain intensity ratings are influenced by pain clusters in different parts of the body [41].

In line with the idea of color or shading encoding, the use of symbols for different types of pain, as illustrated in Figure 5, has been more widely adopted since its inception in 1975 [42,43]. Possible reasons may be that reproduction of color figures were inaccessible and costly, and at that time, medical publications rarely contained color. Symbol-based PDs, on the contrary, could be easily photocopied, interpreted, and presented in black-and-white or grayscale images.

A new element added to the PD in 1988 was the systematic use of arrows to indicate radiating pain. Hildebrandt et al specifically instructed their patients to use arrows to document the area of the pain and the radiation extent [44].

#### Generalizations of the Pain Drawing

The second cluster of conceptual milestones are generalizations made to the *classic PD* over the decades. These include the addition of depth and time dimensions and inclusion of other symptoms and sensations.

One of the major shortcomings of early PDs was their inability to depict the depth of the patient’s pain. Melzack in 1975 used the letter E for external and I for internal to encode depth [42], which is similar to that used by Margoles, who had his patients use a D to distinguish deep from superficial pain [29]. However, the *letter* method only allows for a rough estimation of the actual symptom depth. The first quantitative approach to rating pain depth in PDs was presented more than 20 years later by Jamison et al, who added a transverse section to their 3D manikin, where patients could put a mark to quantify depth as the distance from the surface [45]. Tucker et al expanded this approach in 2014 to calculate depth differences of pain elicited by stimulation of different muscle [35].

In addition to depth, the concept of PDs was also expanded in the third and fourth dimension: The first 3D PDs were acquired by Ghinea et al in 2008 [6]. With their software, patients could manipulate the position of the manikin in all directions before marking their painful region on its body surface. Furthermore, 6 years later, the same group used virtual reality to visualize 3D PD to their patients [46]. Tracking the temporal dynamic of back pain patterns was first accomplished by Gibson and Frank [47]. In a feasibility study, they collected 2-hourly PDs and visual analogue ratings from users of electric wheelchairs finding that pain increased throughout the day in all users and was worst in the neck, back, and buttocks region.

Another PD generalization concerns the inclusion of other sensations, such as paresthesias by Melzack in 1975 [42]. More recently, Bertilson et al introduced the discomfort drawing, where patients mark all areas with pain and any other sort of discomforts such as nonpainful but unpleasant buzzing, tingling, or aching sensations. After completing the drawing, patients write the sort of discomfort next to the drawing using their own words [40].

#### Sex-Specific Aspects

Gender and sex-specific aspects in PDs were largely ignored until Udén and Landin introduced their gender-specific body outlines [31,48]. Before then, all body outlines were either male or relatively androgynous with prominent male appearance. The first report of gender differences in PD outcomes showed that women with neck-shoulder pain tend to draw larger areas and that their PDs are more symmetric than that of men [49]. This finding was later confirmed in a study exploring sex differences in musculoskeletal pain [50]. In the following years, specific body outlines for breast cancer survivors [32] and pregnancy-related lumbopelvic pain [33] emerged.

Finally, in 2016 and almost 30 years after the Udén and Landin paper, Egsgaard et al published the first investigation on qualitative effects of gender-specific body charts reporting that patients believed sex-specific body charts facilitate the communication of pain [37].

### Pain Drawing Analysis Milestones

**Figure 6:**
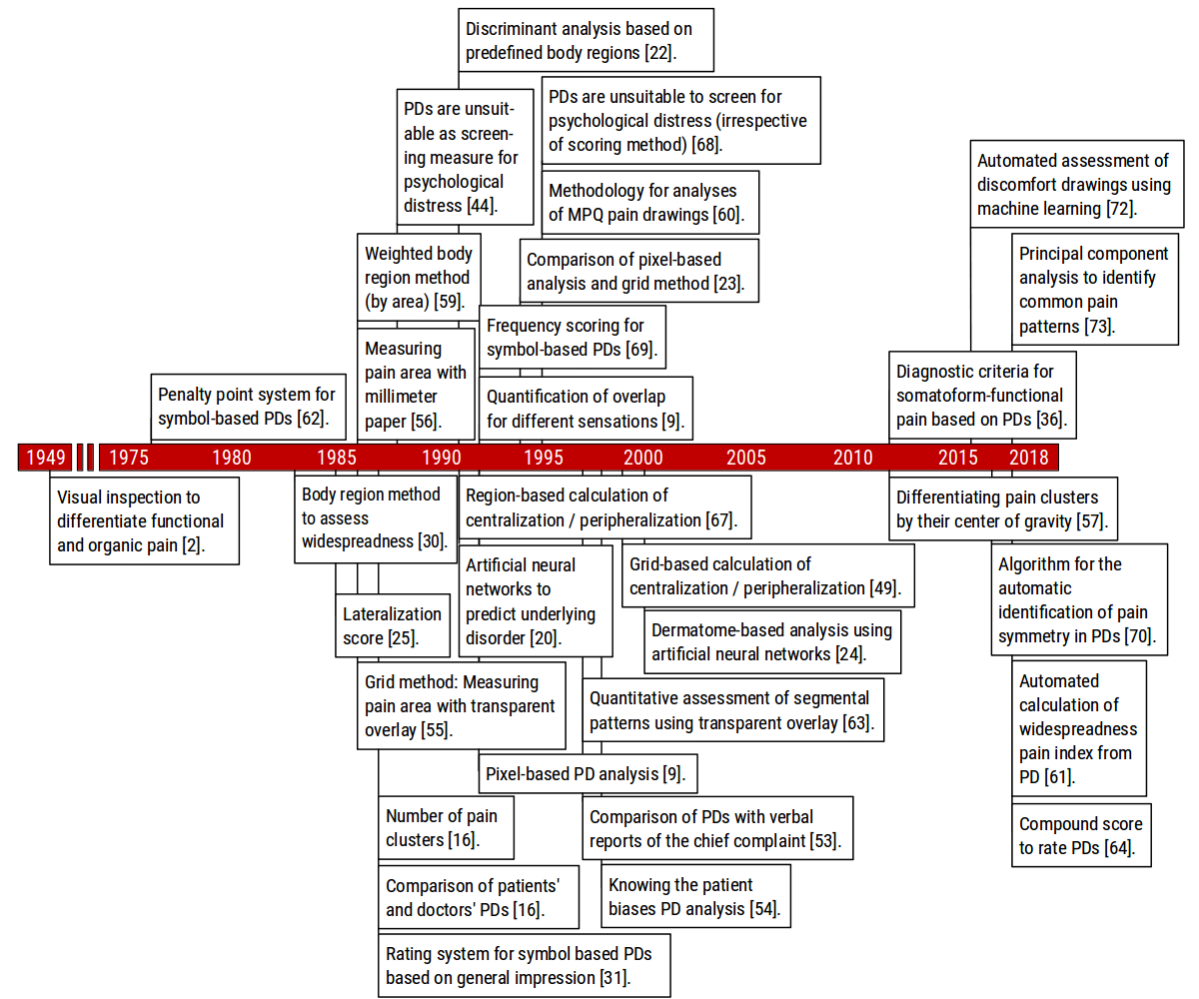
A timeline of methodological milestones contributing to advancements in PD analysis methods showing the golden age from 1985 to around 2000 as well as a renewal of interest in the 2010s. PD: pain drawing; MPQ: McGill pain questionnaire; IPQI: Integrated Pain Quantification Index.

Like all expressions of pain, whether verbal or graphic, PDs need to convey meaningful and useful insight [3]. As this interpretation is not always straightforward, there is a need for interpretation aids that help clinicians draw the right conclusions from a particular PD. As illustrated in Figure 6, the aim to reduce subjectivity in PD analysis sparked a large number of PD-derived measures, rating systems, and diagnostic criteria.

#### Pain Drawing Reporting Style

Although the review is restricted to patient-made PDs, countless examples of PDs made by doctors appear throughout the medical literature [3]. Thus, the question of what differentiates the 2 types of PDs is an important one. In 1987, Cummings and Routan published the first study directly comparing patients’ and doctors’ PDs [15]. Interestingly, the authors presumed but did not confirm doctors’ drawings to be more accurate as they were based upon a physical examination, and thus, patients’ PDs should be compared with them. Similar studies were later published by other groups [51,52].

Another analysis milestone was achieved by comparing patients’ PDs with their verbal reports of the chief pain complaint. The results showed that patients’ verbal descriptions of their chief complaint to a dentist frequently failed to capture and communicate pain located outside of the face region [53]. In this case, the additional pain—may be an important sign of temporomandibular disorders—was captured by using PDs, according to the authors, a prerequisite for initiating adequate treatment [53].

A central topic to the integrity of PDs is the bias of the observer. Here, Reigo et al recommend that the communication of information to be documented in a PD be performed in a blinded fashion. By comparing interobserver agreement in blinded and unblinded doctors, Reigo et al showed that clinical knowledge of the patient appears to introduce a strong bias [54].

#### Measures Derived from Pain Drawings

Measures derived from patients’ PDs can be broadly divided into those that incorporate topographic measures (ie, anatomical knowledge) and those that do not, henceforth called simple measures.

##### Simple Measures

The most common and relatively simple measures obtained from PDs are pain area and extent. Pain area can be defined as the total area marked in a PD, whereas pain extent refers to how many different regions of the body are affected by the pain.

Pain area, as based on pixel counts, is easy to quantify in digitized PDs. However, a similar measure for pen-on-paper drawings requires additional interpretation tools, such as a grid system. Quantitative approaches for the assessment of pain area began in 1986. Gatchel et al applied a transparent overlay onto pen-on-paper drawings that displayed a grid system [55], whereas Fordyce et al used a transparent overlay of millimeter paper to quantify the pain area [56]. These 2 approaches added the area of pain as a new quantitative measure to the (short) list of independent pain assessment outcomes. One year later, Cummings and Routan introduced the number of *distinct pains*, that is, pain clusters as a new measure in their comparison of doctors’ and patients’ PDs [15]. North et al made the first pixel-based analysis of PDs in 1992 [9], where they quantified pain area by pixel count. The same study also introduced the quantification of overlap of different pain sensations, a method used by the authors to determine optimal stimulation settings for their spinal cord stimulators. Two years later, Bryner [23] directly compared the grid system and pixel-based calculation methods, showing that the grid system overestimates pain area. The comparison made clear that the grid system introduces error, and the authors encouraged the adoption of pixel-based measurements. Finally, in 2012, Alonso-Blanco et al showed that the center of gravity is a means to localize pain clusters and, for example, differentiate between referred muscle pain, as demonstrated in myofascial pain and fibromyalgia syndrome [57].

##### Topographic Measures

The most commonly used topographic features of the body in PD analysis are predetermined body regions [58-61] and dermatomes [24,62-64].

In 1983, 3 years before Gatchel et al’s and Fordyce et al’s introduction of quantitative pain area assessment, Toomey et al published a body region method for assessing the total number of pain sites. This method consisted of counting the number of painful body regions using a predefined set of 32 scorable sites located over 7 body areas (head and neck, jaw, chest, abdomen, back, arms and hands, and legs and feet) [30]. It is important to note that the results obtained with this method are not so much a measure of pain area than of pain extent (or widespreadness). The method is very similar to that used for calculating the widespread pain index (WPI), an essential part of the 2010 and 2011 diagnostic criteria for fibromyalgia [65,66]. The WPI was a development independent of PD rating methods per se; however, Shaballout et al created an electronic PD automatically calculating WPI by masking the digital PD with a template of the 19 body regions and counting the nonempty regions [61].

A highly cited modification of the body region method came from Margolis et al in 1986 [59]. The authors published a new body outline with a different set of 45 scorable sites reflecting boundaries of anatomical landmarks. By assigning weights to each of the 45 scorable sites equal to the percentage of body surface, Margolis et al further developed the method to reflect a mixture of pain extent and widespreadness. By comparing their approach to a penalty point method introduced by Ransford et al (see below), the authors found that this method accounted for 56% of the variance in penalty point ratings. These findings suggest that the amount of body surface in pain may hold predictive power for screening patients with psychological distress or dysfunction.

In 1995, Escalante et al applied the body region method to analyze the PD in the McGill pain questionnaire (MPQ) [60]. As no rating system had been available before, they provided a set of scorable body sites adapted to the McGill body outline that could be printed on transparencies and opened the door for epidemiologic PD analyses of the MPQ.

Assessing the dermatomal distribution of pain has been a central part of PD analysis from the very beginning. However, whether or not a given pain pattern looked anatomically meaningful [62] is a debatable question, quantitative methods for pain pattern analysis were not used until 1997. Türp et al applied transparency-based dermatomal maps to distinguish local, regional, and widespread pain based on the exact dermatomal distribution [63]. More recently, Wallace et al introduced dermatome distance measure, defined as the number of segments between the most cranial and the most caudal painful dermatomes, to assess the severity of chronic pain disorders. Together with pain area, intensity, and persistence, the dermatome distance is part of the proposed Integrated Pain Quantification Index (IPQI), a one-dimensional pain score for representing the complex, multidimensional pain experience [64].

Other topographic measures derived from PDs are lateralization and peripheralization indices. Here, Margolis et al [58] were the first to calculate pain lateralization in chronic pain patients. On the basis of a body region approach, left-sided scores are subtracted from right-sided scores, so that positive and negative results indicate a right or left-sided lateralization, respectively [58]. Centralization or peripheralization, that is, a change in the distal-most extent of referred pain toward the lumbar midline or further away from it can be calculated similarly. Donelson et al differentially weighted body regions of the lower body by their distance from the lumbar region; thus, pain in more peripheral areas led to a higher score [67]. Finally, Toomingas used a grid-based approach to calculate the central-peripheral distribution of pain as the mean distance from the central line in a study characterizing neck, shoulder, and upper back pain among the general working population [49].

#### Rating Systems and Diagnostic Criteria

In the study by Palmer in 1949, interpretation of PDs was based on visual inspection, thus, relying solely on a doctors’ experience [2]. Later, researchers applying the visual inspection method have emphasized certain aspects such as dermatomal patterns and symmetry as these criteria were originally proposed by Palmer as a method to differentiate between functional and organic pain. The first semiquantitative rating system to help differentiate between functional and organic pain came from Ransford et al in 1976 [62]. The group aimed to distinguish *organic* low back pain from what today would be called somatization disorder. The rating system assigned penalty points for elements of a PD, such as poor anatomic localization, drawings showing *expansion* or *magnification* of pain (eg, markings outside the outline), explanatory notes, circles or arrows to indicate particularly painful areas, or a tendency toward total body pain. The rating system was widely applied as well as criticized by many groups who were unable to replicate the original results. For instance, Hildebrandt et al showed that PDs as a screening measure for psychological distress were unreliable [44], and by comparing different scoring methods, Parker et al concluded that none of the methods was able to identify distressed patients or differentiate between organic and nonorganic pain patterns [68]. Since the original publication of Ransford’s penalty point method, different modifications have been developed. In 1987, Udén et al noted that many of their patients were circling painful areas, adding explanatory notes, and making markings outside the body outline despite showing otherwise organic pain that responded to treatment. They developed a less quantitative approach based on *general impression* [31]. In addition, 5 years later, Sivik et al published a modification of Ransford’s method replacing some of the more subjective elements by a frequency scoring approach based on the following numbers: different pain types, markings in total, markings outside the body outline, markings with poor anatomical localization, and own markings [69].

More recently, Egloff et al developed diagnostic criteria for somatoform or functional pain by applying strictly quantitative methods of picture analysis [36]. Similar to Udén et al, they found that circle marks and marks outside the body outline are not specific for somatoform pain. In general, PDs with a higher number of marks, typically with symmetric patterns and the presence of long marks (lines), were identified as having a somatoform-functional origin [36]. Most recently, an algorithm for objective classification of symmetric pain patterns for electronic PD was developed and tested in patients with a common knee pain condition known as patellofemoral pain (PFP) [70].

#### Data Mining and Machine Learning Approaches

New possibilities for PD analysis opened up with the introduction of electronic PDs and computer-based analysis programs. Both developments move the potential of PD closer to becoming a tool capable of identifying the underlying cause of specific pain patterns reliably. The first step in this direction was by applying artificial intelligence in the form of ANNs to analyze PDs from patients with low back pain [20]. In the same year, the authors reached another milestone by using discriminant analysis based on predefined body regions to classify PDs into 1 of 5 lumbar spine disorders, with an accuracy of 46.2% (chance level: 20%) [22]. Surprisingly, this was only slightly lower than human expert raters, who reached 51% correct classifications [71]. Several years later, Sanders et al proposed a low back pain triage (degree of urgency) software application. They showed that training an ANN with dermatomal patterns resulted in significantly better classification than when training was performed using simple grid-based PD data [24]. More recently, Zhang et al developed a decision support system using machine learning to automatically assign diagnostic labels to PDs (discomfort drawings) [72]. The latest milestone applied principal component analysis (PCA) and k-means clustering to a relatively large cohort of patients with PFP and revealed 3 mutually independent pain distribution patterns [73]. Although the PCA study focused on knee pain, the results further support the utility of using ANN for diagnostic applications, whereas the results stemming from other machine learning methods suggest that these methods in combination may help identify and clarify underlying drivers of many (painful) diseases and syndromes.

### Pain Drawing Visualization Milestones

**Figure 7:**
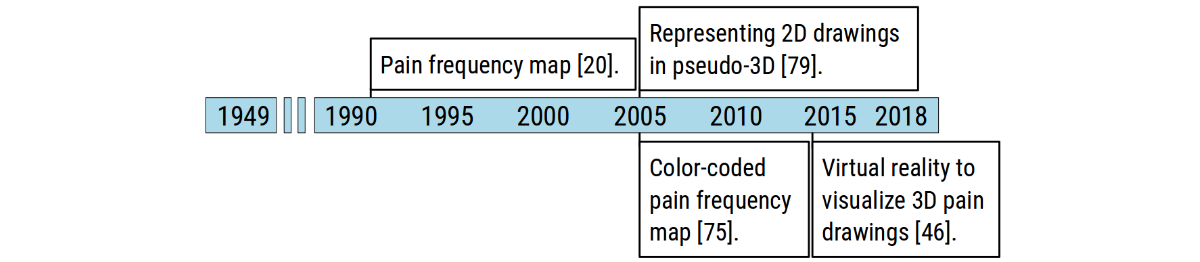
Timeline of methodological milestones in the area of pain drawing visualization. 3D: three-dimensional; 2D: two-dimensional.

PDs are an instrument to visualize, document, and explore otherwise difficult descriptions of the pain experience. The last of the 4 main areas of methodological advancement is, therefore, a summary of milestones for visualizing data captured by using PDs and the results derived from them (Figure 7).

One of the most common ways of presenting statistical group results in PD studies is pain frequency maps. These maps show the distribution of pain for a select group (eg, patients), and the first was published in 1991 by Mann and Brown [20]. The pain frequency map used points for each pain mark from each PD and gave a rough visual impression of the most common pain locations and distribution in patients with spinal stenosis, herniated disc, and other underlying disorders. Other early and very different maps came from the groups of Türp et al, Slipman et al, and Svensson et al, who used bar charts [74], grayscale grids [75], and overlays of the tracings of each person’s pain map [76] to represent pain frequency. Slipman et al later published the first color-coded pain-frequency map showing the dermatomal distribution of referred pain evoked by stimulating individual cervical discs [77]. In the PCA study by Boudreau et al, the pain frequency map represented the raw PD and was then filtered to differentiate more clearly the most common shape of each pain distribution pattern on and around the knee [73].

An interesting analogy exists between PDs and geographic maps as reported by Ghinea et al, who in 2002 suggested that geographic information systems are a suitable technical solution for storing and analyzing digitized PDs as well [78].

Finally, several groups developed ways to improve visualization of PDs, notably Hwang et al, who represented results of 2D drawings on a pseudo-3D body template [79] and Spyridonis et al, who used virtual reality to visualize 3D PDs to their patients [46].

## Discussion

### Principal Findings

In this review, we have compiled a historical timeline detailing several methods for analyzing and visualizing data captured using PDs as well as conceptual steps for improving the applicability of PDs for basic and clinical research. A majority of the milestones revolve around PD analysis and interpretation. The systematic literature review revealed continuous developments along different lines of progression, namely *PD acquisition*, *conception of PDs*, *PD analysis*, and *PD visualization*. In combination, these developments result in a more sophisticated PD since the original introduction. The future of PDs will depend on the utilization and adoption of the information into research and clinical settings. Advances in visualization of the information acquired by PDs may help facilitate this process, as this appears to be the most recent line of progression emerging in PD history.

### A Toolbox for Clinic and Science

The conceptual developments of PDs mainly focused on improving the body templates to capture a deeper understanding of the pain experience and to better match the individual. These improvements revolve around core elements of the PD and include sex-specific body templates and encodings of intensity or the quality of pain. Altogether, these core elements can be viewed as a toolbox offering researchers and clinicians a number of options. Some of these core elements were already proposed by Palmer’s groundbreaking publication [2], whereas others represent recent additions. A primary core element is the choice of a body template on which the drawing is to be made and of which a growing number of different versions exist (Figure 3). Encoding of sensations is a core element that manifests in many different versions, such as different symbols [43] or colors [29,39], expressing pain intensity by saturation [40], rating pain intensity for individual clusters [41], and indicating pain radiation by arrows [44]. Further supplemental PD elements are methods and measures to assess the depth of pain either by the *distance to body center* method [35,45] or by a simple binary rating using the letters E for external and I for internal [42]. An intermediate approach is to let patients choose among descriptions *on the skin*, *beneath the skin, muscle*, *organ*, and *bone* [80].

Overall, these core elements can be combined when tailoring a PD approach to a particular clinical or scientific need. To date, it is unclear which version of the core elements, body templates, and encoding of sensations is best. At this time, there is no dominant template or method, and this will contribute to a lack of standardization. Furthermore, PDs can be used to assess more than just pain, for example, discomfort drawings [40], general symptom drawings [80] or sensation drawings as evidenced by the recent application of PDs in studies on emotions [81-83], the placebo effect [84], or acupuncture [85,86]. This means the encoding of sensations may continue to develop in this area as the applicability of PDs expands.

### How to Analyze a Pain Drawing

There are a significant number of PD analysis milestones. Many of them originating from pen-and-paper methods and then progressing into opportunities created by the introduction of PCs, tablets, and smartphones. The methods range from how to calculate pain area, extent, and widespreadness to encoding the PD as a grid system for ANN training. The analysis techniques are becoming sophisticated and require multidisciplinary teams extending from the clinic to mathematics and computer science. The PDs are similar to any other image that would be utilized in computer vision, such as those capable of identifying and discriminating between dog and cat. Methodological advances in PD analysis have resulted in several methods for quantifying the 3 main aspects of pain captured by the PD: pain intensity, localization, and distribution. Quantitative information on these aspects is well suited to complement other pain assessments such as questionnaires or analogue scales. Analytical methods include simple measures, such as pain area [55,56] or the number of clusters [15], topographic measures, such as segmental involvement [62,63] or widespreadness [61], and compound measures, such as the IPQI [64], that combine simple and topographic measures in a single one-dimensional score. More PD-derived measures will likely be developed, and their usefulness tested with the broader application of digital image analysis tools.

### Need for Standardization

When reviewing and assembling the milestones, a common observation was a general lack of standards for using PDs. This concerns almost all critical methodological aspects. As a result, comparison of different study results is often complicated and sometimes impossible.

One of the main problems that has been pointed out as early as 1980 [29] is the lack of a standard body template for PD studies. Although many may argue that an arm is an arm irrespective of the exact body template used, we need to ask ourselves how much a PD body template biases the results by showing a muscular or skinny arm. Furthermore, many body templates differ in posture, and only some include lateral views. We acknowledge that there are special purposes such as assessing pain in pregnancy that necessitate the development of new body templates (see our discussion of milestones). For all other applications, however, it would be highly desirable that the community adopts a common body template or restricts itself to a minimal number of different templates. The question, however, is which one? To date, high-quality templates should be usable for both pen-and-paper and digital PDs [5,23], show all relevant body regions [29,30], be sex-specific [31], and ideally come with information on dermatomes [63] and other topographic regions [30,59]. As copyright is a common obstacle, the body template and dermatome schema provided by Neubert et al [61,87] has been made available under an open license, making the template free to use and accessible (asking permission is not required) [87]. We encourage publishers and the community to follow this example and release the body templates or start using templates that are openly available. Naturally, this also includes the emerging area of 3D or pseudo-3D body templates, where some realistic templates are available for research purposes [88,89] or even under an open license [90,91].

The second area in need of standardization is the instructions given to a patient before creating a PD. In our opinion, this aspect has not received enough attention in the literature, which has led to much confusion. One famous example is the drawing of symbols outside of the body template, the circling of painful areas, and the adding of explanatory notes by the patient, which some researchers consider signs of a somatization disorder [62,69], whereas others see them as perfectly reasonable ways to express pain [31]. In our experience, commonly used instructions such as “Describe your pain on the pain drawing” or “Mark your symptoms on this figure” lack the specificity necessary to achieve a consistent drawing style in patients. Therefore, efforts should be made to determine the optimal set of instructions. One possibility may be to convey instructions in a graphical form [80,92]. Furthermore, when replicating a study from another group, researchers should use the same PD instructions used by that group. In the clinic, a joint discussion of the completed PD by the patient and the physician can help to avoid misunderstandings [2,93].

### Limitations

Our approach to review the literature has several limitations. First, our selection of *PD acquisition*, *conception of PDs*, *PD analysis*, and *PD visualization as* categories to document advancements and individual milestones may inadvertently bias reporting. Second, the literature search primarily sourced papers assessing and evaluating PDs as primary instruments of measure and thus may miss publications outlining advancements as secondary or exploratory PD outcomes. Moreover, publications outlining acquisition and analysis methods of PDs were often insufficient to fully compare and differentiate different approaches. Thus, we may have also overlooked papers that have introduced a particular advancement but not explicitly mentioned it. Third, our literature search identified relevant publications by the search terms appearing in the title or abstract. Thus, any publications on PDs not mentioning these terms in the title or abstract have not been included. Furthermore, internet-based searches, in general, are prone to neglect publications from the preinternet era that are not digitized. We have tried to mitigate this limitation by checking the reference lists of all milestone papers before 1990, but we may still have overlooked important contributions.

### How Digital Technology May Shape the Future of Pain Drawings

Digital technologies have had a significant influence on the evolution of PD methodology and will continue to do so. Although the first digital PDs in the early 90s [9,20] were still much more cumbersome than filling out pen-and-paper counterparts, digital PD acquisition has come a long way. Today, touch screens and digital pens are replacing keyboards and the computer mouse for data entry, whereas modern software apps work essentially like digital pen-and-paper platforms [5,7,87,94]. Furthermore, the latest available systems actively guide their users through the drawing process and thus improve PD quality. For example, guides help patients to conform to a particular drawing style (eg, by automatically filling closed shapes or restricting the drawable area to the body template). Some digital systems are even capable of calculating PD-derived measures in real time to aid diagnosis. These capabilities were initially developed to reduce research time and allow for more accurate assessments of the drawn pain area(s). However, the automatic calculations and visualization techniques provide a glimpse of new possibilities for integrating such information into busy workflows (clinic or research alike). Thus, it is an exciting prospect that such systems will become widely disseminated in the near future, and their clinical and scientific potential will be realized.

An area that has also significantly gained from digital technologies is PD analysis. Here, computer-based methods offer the possibility to analyze pain patterns in ever greater detail. Although digitally acquired PDs are advantageous for such analyses, they are by no means a prerequisite, as evidenced by numerous papers applying elaborate analyses to scanned or otherwise digitized pen-and-paper PDs [24,32,64,78,79]. Digital image processing also allows for digitization and analysis of extensive collections of pen-and-paper PDs (eg, as seen in the study by Wallace et al [64]), treasures which may be buried in medical archives).

However, the new technological possibilities also raise the question if specific popular methods, such as symbol-based PDs, grid-based methods [34,55], the counting of clusters [15] and scoring of body sites [30,59], are still in step with the times. They all reflect the limitations of the predigital age when photocopies were black and white, journal articles contained no color figures, and all PD scoring had to be done by hand. As a result, symbol-based PDs became popular as they could be reproduced in black and white and analyzed quickly by counting the different symbols, even though they were less intuitive for patients and had their spatial resolution limited by the size of the symbols, thus affecting all further analyses. In the age of digital PDs, however, these limitations no longer exist. Thus, instead of clinging to established yet outdated methods, we should embrace the new possibilities of the digital age. Patients can draw their pain patterns in full color at a resolution similar to that of pen-and-paper drawings and add information on intensity, depth, or any other relevant attribute in an iterative way. The final PDs can be archived and shared in full color either electronically or in printed form. Instead of reducing data size to a level that can be handled without a computer, measures derived from digital PDs can utilize all available data down to the pixel level and apply mathematical transformations of high complexity [64,73].

However, even the most informative measures, such as pain area, can never replace the PD itself. Although these simple measures are crucial for quantification and statistical analyses, there is an advantage by allowing our inherent ability to process and recognize patterns visually, thus individual examples of actual PDs should also be included in all future publications. Indeed, the ability to efficiently communicate pain is a primary advantage of PDs.

### Conclusions

The PD as a clinical and research tool has undergone significant methodological development in the last 70 years and will continue to do so in the future. PDs capture many aspects of the subjective pain experience and have applications well beyond the pain specialty. Recent technological advancements, together with the versatility of the PD, have led to renewed interest in the past decade. Thanks to the transition from pen on paper to digital, we may soon see the dawn of a golden age of PDs.

## Appendix

Multimedia Appendix 1 List of methodological milestones.

## Abbreviations

2D: two-dimensional
3D: three-dimensional
ANN: artificial neural network
IPQI: Integrated Pain Quantification Index
MPQ: McGill pain questionnaire
PC: personal computer
PCA: principal component analysis
PD: pain drawing
PFP: patellofemoral pain
WPI: widespread pain index
