# Supplementary material for: From Paper to Digital Applications of the Pain Drawing: Systematic Review of Methodological Milestones
Source: JMIR Mhealth Uhealth. 2019 Sep 5;7(9):e14569. doi: 10.2196/14569 (PMC6753689; doi:10.2196/14569)
Supplement: Multimedia Appendix 1 [file mhealth_v7i9e14569_app1.pdf]

This is a Multimedia Appendix to a full manuscript published in the J Med Internet Res. For full copyright and citation information see http://dx.doi.org/10.2196/mhealth.14569.

Multimedia Appendix

List of methodological milestones

Publication	Area of advancement				Description of methodological milestones
	Conceptual	Acquisition	Analysis	Visualization	
Palmer [2]	—	x	x	—	First known description of pen-on-paper pain drawings (PDs). Visual inspection approach for differentiation of functional and organic pain based on symmetry.
Melzack [19]	x	—	—	—	Encoding depth by letters E for external and I for internal. Generalization of PD to other sensations (eg, paresthesia).
Mooney et al [20]	x	—	—	—	Use of symbols for different types of pain.
Ransford et al [21]	—	—	x	—	Penalty point system for symbol-based PDs to diagnose somatization disorders from PD.
Margoles et al [22]	x	x	—	—	Common body outline with 4 views with the goal of standardization. Use of colors for different kinds of pain.
Toomey et al [23]	—	x	x	—	Body region method to assess total sites of pain as a measure for widespreadness. Separate body outline for headaches.
Margolis et al [24]	—	—	x	—	Lateralization score by subtracting left side score from right side score.
Fordyce et al [25]	—	—	x	—	Using millimeter paper to measure pain area.
Gatchel et al [26]	—	—	x	—	Grid method using transparent overlay to measure pain area in the body outline and surrounding space.
Margolis et al [27]	—	—	x	—	Weighted body region method (by area).
Cummings et al [16]	x	—	x	—	Comparison of patients' and doctors' PDs; number of clusters.
Udén et al [28]	x	x	x	—	Sex-specific body outlines. Rating system for symbol-based PDs based on general impression.
Hildebrandt et al [29]	x	—	x	—	Arrows to indicate pain radiation. Showing major limitations of PDs as screening measure for psychological distress.
Donelson et al [30]	—	—	x	—	Region-based calculation of centralization and peripheralization.

This is a Multimedia Appendix to a full manuscript published in the J Med Internet Res. For full copyright and citation information see http://dx.doi.org/10.2196/mhealth.14569.

Mann et al [31]	—	x	x	x	Pain frequency map. Analysis using artificial neural networks to predict underlying disorder. PD acquisition via mouse on computer screen.
Mann et al [32]	—	x	x	—	Discriminant analysis based on predefined body regions. Digitization of pen-on-paper PD using mouse on computer screen.
North et al [9]	—	x	x	—	Pixel-based PD analysis. Quantification of overlap for different sensations. PD acquisition via graphics tablet on computer screen.
Sivik et al [34]	—	—	x	—	Frequency scoring for symbol-based PDs.
Bryner [35]	—	x	x	—	Comparison of pixel-based analysis and grid method. Digitization of pen-on-paper PD using graphics tablet.
Escalante et al [36]	—	—	x	—	Methodology for analyses of McGill pain questionnaire PDs.
Parker et al [37]	—	—	x	—	PDs as screening measure for psychological distress have major limitations irrespective of scoring method.
Türp et al [38]	x	—	x	—	Quantitative comparison of PDs with verbal reports of the chief complaint.
Türp et al [39]	—	—	x	—	Quantitative assessment of dermatomal patterns using transparent overlay.
Aló et al [4]	—	x	—	—	PD acquisition via pen on computer touch screen.
Reigo et al [40]	—	—	x	—	Assessing bias in PD analysis caused by clinical knowledge of the patient.
Toomingas [41]	x	—	x	—	Grid-based calculation of centralization and peripheralization. Quantitative analysis of sex-related effects on PD outcomes.
Sanders et al [42]	—	x	x	—	Dermatome-based analysis using artificial neural networks. Digitization of pen-on-paper PD using video camera.
Türp et al [43]	x	—	—	—	Pain intensity ratings of individual clusters.
Ghinea et al [44]	x	—	x	—	Using a geographic information system exploiting similarities of PDs and geographical maps.
Bertilson et al [45]	x	—	—	—	Generalization of PD to discomfort drawing. Using saturation to encode pain intensity.
Masferrer et al [46]	x	—	—	—	First detailed analysis of colored PDs.
North et al [33]	—	x	—	—	PD acquisition on a tablet personal computer (PC).
Hwang et al [47]	—	—	—	x	Representing results of two-dimensional (2D) drawings in pseudo-three-dimensional (3D).
Gibson et al [48]	x	—	—	—	Assessing temporal changes in PDs.
Slipman et al [49]	—	—	—	x	Color-coded pain-frequency map.
Ghinea et al [6]	x	x	—	—	First 3D PD.
Jud et al [50]	—	x	—	—	Body outline for breast pain.
Elson et al [51]	—	x	—	—	Photographic (partial) body outline.

Jamison et al [52]	x	—	—	—	Quantitative approach to rating pain depth as distance from the surface.
Egloff et al [53]	—	—	x	—	Diagnostic criteria for somatoform-functional pain based on PDs.
Pierce et al [54]	—	x	—	—	Pregnant body outline.
Alonso-Blanco et al [55]	—	—	x	—	Differentiating pain clusters by their center of gravity.
Jaatun et al [7]	—	x	—	—	Detailed comparison of PDs acquired by tablet PC versus pen-on-paper and laptop-based PDs.
Southerst et al [10]	—	x	—	—	Assessment of interrater reliability and intermethod reliability of digital and paper PDs.
Spyridonis et al [56]	x	—	—	x	Use of virtual reality to visualize 3D PDs.
Tucker et al [57]	x	—	—	—	Calculation depth difference of pain from different sources.
Jaatun et al [58]	—	x	—	—	Design guidelines for PD software apps for patients who are frail, very sick, or have cognitive impairments.
Egsgaard et al [59]	x	x	—	—	Qualitative effects gender-specific body charts. Comparison of 2D and (pseudo-)3D body outlines.
Zhang et al [60]	—	—	x	—	Automated assessment of discomfort drawings using machine learning.
Boudreau et al [61]	—	—	x	—	Algorithm for the automatic identification of pain symmetry in PDs.
Boudreau et al [62]	—	—	x	—	Principal component analysis to identify common pain patterns.
Shaballout et al [63]	—	—	x	—	Automated calculation of pain widespreadness (widespread pain index).
Wallace et al [64]	—	—	x	—	Compound score (Integrated Pain Quantification Index) to rate PDs based on number of noncontiguous painful areas, dermatome distance, and pain intensity.

References

2. Palmer H. Pain charts; a description of a technique whereby functional pain may be diagnosed from organic pain. The New Zealand medical journal 1949;48:187-213.

4. Alo K, Yland M, Kramer D, Charnov J, Redko V. Computer assisted and patient interactive programming of dual octrode spinal cord stimulation in the treatment of chronic pain. Neuromodulation: Journal of the International Neuromodulation Society 1998;1:30-45. PMID:22150884

6. Ghinea G, Spyridonis F, Serif T, Frank A. 3-D pain drawings-mobile data collection using a PDA. IEEE transactions on information technology in biomedicine: a publication of the IEEE Engineering in Medicine and Biology Society 2008;12:27-33. PMID:18270034
7. Jaatun E, Haugen D, Dahl Y, Kofod-Petersen A. Proceed with Caution: Transition from Paper to Computerized Pain Body Maps. Procedia Comput Sci 2013;21:398-406. DOI: 10.1016/j.procs.2013.09.052
9. North R, Nigrin D, Fowler K, Szymanski R, Piantadosi S. Automated 'pain drawing' analysis by computer-controlled, patient-interactive neurological stimulation system. Pain 1992;50:51-57. PMID:1381071
10. Southerst D, Stupar M, Côté P, Mior S, Stern P. The reliability of measuring pain distribution and location using body pain diagrams in patients with acute whiplash-associated disorders. J Manipulative Physiol Ther 2013;36:395-402. PMID:23891480
16. Fortin J, Aprill C, Ponthieux B, Pier J. Sacroiliac joint: pain referral maps upon applying a new injection/arthrography technique. Part II: Clinical evaluation. Spine 1994;19:1483-1489. PMID:7939979
19. Melzack R. The McGill Pain Questionnaire: Major properties and scoring methods. Pain 1975;1:277-299. PMID:1235985
20. Mooney V, Cairns D, Robertson J. A system for evaluating and treating chronic back disability. The Western Journal of Medicine 1976;124:370-376. PMID:132014
21. Ransford A, Cairns D, Mooney V. The Pain Drawing as an Aid to the Psychologic Evaluation of Patients With Low-Back Pain. Spine 1976;1:127-134.
22. Margoles M. Letter to the editor. Pain 1980;8:115-117.
23. Toomey T, Gover V, Jones B. Spatial distribution of pain: a descriptive characteristic of chronic pain. Pain 1983;17:289-300. PMID:6657289
24. Margolis R, Krause S, Tait R. Lateralization of chronic pain. Pain 1985;23:289-293. PMID:4069726
25. Fordyce W, Brockway J, Bergman J, Spengler D. Acute back pain: a control-group comparison of behavioral vs traditional management methods. J Behav Med 1986;9:127-140. PMID:2940370
26. Gatchel R, Mayer T, Capra P, Diamond P, Barnett J. Quantification of lumbar function. Part 6: The use of psychological measures in guiding physical functional restoration. Spine 1986;11:36-42. PMID:2939568
27. Margolis R, Tait R, Krause S. A rating system for use with patient pain drawings. Pain 1986;24:57-65. PMID:2937007

This is a Multimedia Appendix to a full manuscript published in the J Med Internet Res. For full copyright and citation information see http://dx.doi.org/10.2196/mhealth.14569.

28. Udén A, Landin L. Pain drawing and myelography in sciatic pain. Clin Orthop 1987;216:124-130. PMID:3815937

29. Hildebrandt J, Franz C, Choroba-Mehnen B, Temme M. The use of pain drawings in screening for psychological involvement in complaints of low-back pain. Spine 1988;13:681-685. PMID:2972073

30. Donelson R, Grant W, Kamps C, Medcalf R. Pain response to sagittal end-range spinal motion. A prospective, randomized, multicentered trial. Spine 1991;16:206.

31. Mann N, Brown M. Artificial intelligence in the diagnosis of low back pain. The Orthopedic Clinics of North America 1991a;22:303-314. PMID:1830700

32. Mann N, Brown M, Enger I. Statistical diagnosis of lumbar spine disorders using computerized patient pain drawings. Comput Biol Med 1991b;21:383-397. PMID:1838724

33. North R, Calkins S, Campbell D, Sieracki J, Piantadosi S, Daly M, Dey P, Barolat G. Automated, patient-interactive, spinal cord stimulator adjustment: a randomized controlled trial. Neurosurgery 2003;52:580. PMID:12590681

34. Sivik T, Gustafsson E, Olsson K. Differential diagnosis of low-back pain patients: A simple quantification of the pain drawing. Nord J Psychiatry 1992;46:55-62. DOI: 10.3109/08039489209106183

35. Bryner P. Extent measurement in localised low-back pain: a comparison of four methods. Pain 1994;59:281-285. PMID:7892026

36. Escalante A, Lichtenstein M, White K, Rios N, Hazuda H. A method for scoring the pain map of the McGill Pain Questionnaire for use in epidemiologic studies. Aging 1995;7:358-366. PMID:8719602

37. Parker H, Wood P, Main C. The use of the pain drawing as a screening measure to predict psychological distress in chronic low back pain. Spine 1995;20:236-243. PMID:7716631

38. Türp J, Kowalski C, Stohler C. Temporomandibular disorders--pain outside the head and face is rarely acknowledged in the chief complaint. The Journal of Prosthetic Dentistry 1997a;78:592-595. PMID:9421788

39. Türp J, Kowalski C, Stohler C. Greater disability with increased pain involvement, pain intensity and depressive preoccupation. European Journal of Pain (London, England) 1997b;1:271-277. PMID:15102392

40. Reigo T, Tropp H, Timpka T. Pain drawing evaluation--the problem with the clinically biased surgeon. Intra- and interobserver agreement in 50 cases related to clinical bias. Acta Orthop Scand 1998;69:408-411. PMID:9798452

41. Toomingas A. Characteristics of pain drawings in the neck-shoulder region among the working population. Int Arch Occup Environ Health 1999;72:98-106. PMID:10197481

42. Sanders N, N. Horace Mann II. Automated scoring of patient pain drawings using artificial neural networks: efforts toward a low back pain triage application. Comput Biol Med 2000;30:287-298. PMID:10913774

43. Türp J, Kowalski C, Stohler C. Generic pain intensity scores are affected by painful comorbidity. J Orofac Pain 2000;14:47-51. PMID: 11203737

44. Ghinea G, Gill D, Frank A, de Souza L. Using geographical information systems for management of back-pain data. J Manag Med 2002;16:219-237. PMID:12211347

45. Bertilson B, Grunnesjö M, Strender L. Reliability of clinical tests in the assessment of patients with neck/shoulder problems-impact of history. Spine 2003;28:2222-2231. PMID: 14520035

46. Masferrer R, Prendergast V, Hagell P. Colored pain drawings: Preliminary observations in a neurosurgical practice. Eur J Pain 2003;7:213-217. PMID:12725843

47. Hwang M, Kang Y, Kim D. Referred pain pattern of the pronator quadratus muscle. Pain 2005;116:238-242. PMID:15964683

48. Gibson J, Frank A. Pain experienced by electric-powered chair users: a pilot exploration using pain drawings. Physiotherapy Research International 2005;10:110-115. PMID: 16146328

49. Slipman C, Plastaras C, Palmitier R, Huston C, Sterenfeld E. Symptom provocation of fluoroscopically guided cervical nerve root stimulation. Are dynatomal maps identical to dermatomal maps?. Spine 1998;23:2235-42. PMID: 9802168

50. Jud S, Fasching P, Maihöfner C, Heusinger K, Loehberg C, Hatko R, Rauh C, Bani H, Lux M, Beckmann M, Bani M. Pain perception and detailed visual pain mapping in breast cancer survivors. Breast Cancer Res Treat 2010;119:105-110. PMID:19641989

51. Elson D, Jones S, Caplan N, Stewart S, Gibson A, Kader D. The photographic knee pain map: locating knee pain with an instrument developed for diagnostic, communication and research purposes. The Knee 2011;18:417-423. PMID: 20850976

52. Jamison R, Washington T, Gulur P, Fanciullo G, Arscott J, McHugo G, Baird J. Reliability of a preliminary 3-D pain mapping program. Pain medicine 2011;12:344-351. PMID:21276186

53. Egloff N, Cámara R, von Känel R, Klingler N, Marti E, Ferrari M. Pain drawings in somatoform-functional pain. BMC musculoskeletal disorders 2012;13:257. PMID: 21276186

54. Pierce H, Homer C, Dahlen H, King J. Pregnancy-related lumbopelvic pain: listening to Australian women. Nursing Research and Practice 2012;2012:387428. PMID: 22685643
55. Alonso-Blanco C, Fernández-de-las-Peñas C, de-la-Llave-Rincón AI, Zarco-Moreno P, Galán-del-Río F, Svensson P. Characteristics of referred muscle pain to the head from active trigger points in women with myofascial temporomandibular pain and fibromyalgia syndrome. The Journal of Headache and Pain 2012;13:625-637. PMID: 22935970
56. Spyridonis F, Hansen J, Grønli T, Ghinea G. PainDroid: An android-based virtual reality application for pain assessment. Multimedia Tools and Applications 2014;72:191-206. DOI: 10.1007/s11042-013-1358-3
57. Tucker K, Fels M, Walker S, Hodges P. Comparison of location, depth, quality, and intensity of experimentally induced pain in 6 low back muscles. The Clinical journal of pain 2014;30:800-808. PMID:25098553
58. Jaatun E, Haugen D, Dahl Y, Kofod-Petersen A. Designing a reliable pain drawing tool: Avoiding interaction flaws by better tailoring to patients' impairments. Personal and Ubiquitous Computing 2015;19:635-648. DOI: 10.1007/s00779-015-0850-3
59. Egsgaard L, Christensen T, Petersen I, Bronnum D, Boudreau S. Do Gender-Specific and High-Resolution Three Dimensional Body Charts Facilitate the Communication of Pain for Women? A Quantitative and Qualitative Study. JMIR human factors 2016;3:e19. PMID:27440737
60. Zhang C, Kjellstrom H, Ek C, Bertilson B. Diagnostic Prediction Using Discomfort Drawings with IBTM. PMLR 2016;56:226-238.
61. Boudreau S, Kamavuako E, Rathleff M. Distribution and symmetrical patellofemoral pain patterns as revealed by high-resolution 3D body mapping: a cross-sectional study. BMC Musculoskeletal Disorders 2017;18(1):160. PMID: 28420379
62. Boudreau S, Royo A, Matthews M, Graven-Nielsen T, Kamavuako E, Slabaugh G, Thorborg K, Vicenzino B, Rathleff M. Distinct patterns of variation in the distribution of knee pain. Scientific Reports 2018;8(1):16522. PMID: 30410031.
63. Shaballout N, Aloumar A, Neubert T, Dusch M, Beissner F. Digital Pain Drawings Can Improve Doctors' Understanding of Acute Pain Patients: Survey and pain drawing analysis. JMIR Mhealth Uhealth 2019;7(1):e11412. PMID:30632970

This is a Multimedia Appendix to a full manuscript published in the J Med Internet Res. For full copyright and citation information see http://dx.doi.org/10.2196/mhealth.14569.

64. Wallace M, North J, Grigsby E, Kapural L, Sanapati M, Smith S, Willoughby C, McIntyre P, Cohen Set al. An Integrated Quantitative Index for Measuring Chronic Multisite Pain: The Multiple Areas of Pain (MAP) Study. Pain Medicine. in press. PMID: 29474648
